# The oncogenic role of the In1-ghrelin splicing variant in prostate cancer aggressiveness

**DOI:** 10.1186/s12943-017-0713-9

**Published:** 2017-08-29

**Authors:** Daniel Hormaechea-Agulla, Manuel D. Gahete, Juan M. Jiménez-Vacas, Enrique Gómez-Gómez, Alejandro Ibáñez-Costa, Fernando L-López, Esther Rivero-Cortés, André Sarmento-Cabral, José Valero-Rosa, Julia Carrasco-Valiente, Rafael Sánchez-Sánchez, Rosa Ortega-Salas, María M. Moreno, Natia Tsomaia, Steve M. Swanson, Michael D. Culler, María J. Requena, Justo P. Castaño, Raúl M. Luque

**Affiliations:** 1 Maimonides Institute of Biomedical Research of Cordoba (IMIBIC), Córdoba, Spain; 2 Department of Cell Biology, Physiology and Immunology, University of Córdoba, Córdoba, Spain; 3 Reina Sofia University Hospital (HURS), Córdoba, Spain; 4 CIBERobn, Córdoba, Spain; 5 ceiA3, Córdoba, Spain; 6 Urology Service, HURS/IMIBIC, Córdoba, Spain; 7 Anatomical Pathology Service, HURS/IMIBIC, Córdoba, Spain; 8 School of Pharmacy, University of Wisconsin-Madison, Madison, WI USA; 9 IPSEN Bioscience, Cambridge, MA USA

**Keywords:** Ghrelin-system, In1-ghrelin variant, Prostate cancer, Aggressiveness

## Abstract

**Background:**

The Ghrelin-system is a complex, pleiotropic family composed of several peptides, including native-ghrelin and its In1-ghrelin splicing variant, and receptors (GHSR 1a/b), which are dysregulated in various endocrine-related tumors, where they associate to pathophysiological features, but the presence, functional role, and mechanisms of actions of In1-ghrelin splicing variant in prostate-cancer (PCa), is completely unexplored. Herein, we aimed to determine the presence of key ghrelin-system components (native-ghrelin, In1-ghrelin, GHSR1a/1b) and their potential pathophysiological role in prostate cancer (PCa).

**Methods:**

In1-ghrelin and native-ghrelin expression was evaluated by qPCR in prostate tissues from patients with high PCa-risk (*n* = 52; fresh-tumoral biopsies), and healthy-prostates (*n* = 12; from cystoprostatectomies) and correlated with clinical parameters using Spearman-test. In addition, In1-ghrelin and native-ghrelin was measured in plasma from an additional cohort of PCa-patients with different risk levels (*n* = 30) and control-healthy patients (*n* = 20). In vivo functional (proliferation/migration) and mechanistic (gene expression/signaling-pathways) assays were performed in PCa-cell lines in response to In1-ghrelin and native-ghrelin treatment, overexpression and/or silencing. Finally, tumor progression was monitored in nude-mice injected with PCa-cells overexpressing In1-ghrelin, native-ghrelin and empty vector (control).

**Results:**

In1-ghrelin, but not native-ghrelin, was overexpressed in high-risk PCa-samples compared to normal-prostate (NP), and this expression correlated with that of PSA. Conversely, GHSR1a/1b expression was virtually absent. Remarkably, plasmatic In1-ghrelin, but not native-ghrelin, levels were also higher in PCa-patients compared to healthy-controls. Furthermore, In1-ghrelin treatment/overexpression, and to a much lesser extent native-ghrelin, increased aggressiveness features (cell-proliferation, migration and PSA secretion) of NP and PCa cells. Consistently, nude-mice injected with PC-3-cells stably-transfected with In1-ghrelin, but not native-ghrelin, presented larger tumors. These effects were likely mediated by ERK1/2-signaling activation and involved altered expression of key oncogenes/tumor suppressor genes. Finally, In1-ghrelin silencing reduced cell-proliferation and PSA secretion from PCa cells.

**Conclusions:**

Altogether, our results indicate that In1-ghrelin levels (in tissue) and circulating levels (in plasma) are increased in PCa where it can regulate key pathophysiological processes, thus suggesting that In1-ghrelin may represent a novel biomarker and a new therapeutic target in PCa.

**Electronic supplementary material:**

The online version of this article (doi:10.1186/s12943-017-0713-9) contains supplementary material, which is available to authorized users.

## Background

Prostate cancer (PCa) is the most frequently diagnosed cancer and the second leading cause of cancer-associated mortality in the male population worldwide [1]. Unfortunately, PCa is a complex and heterogeneous cancer type that progresses, mostly unnoticed, from slow-growing, tissue-confined lesions to highly aggressive and metastatic forms, through a process wherein the endocrine regulation plays a pivotal role [2–4]. Thus, it is mandatory to gain a deeper knowledge of PCa pathophysiology and to better define tumor behavior (from proliferation to metastasis mechanisms), in order to identify new molecular diagnostic/prognostic markers and to provide clues for novel therapeutic targets. Indeed, although the vast majority of studies have been focused on the role played by androgens on PCa development/progression and their therapeutic implications [5, 6], other endocrine systems have been also associated with PCa tumorigenesis, including estrogens, LH/FSH, IGF-I, somatostatin, etc. [7, 8].

In this sense, it is known that the majority of the components of the ghrelin-family, a key endocrine/metabolic regulatory system, are locally expressed in the prostate [2, 9]. Specifically, native-ghrelin, the endogenous ligand for the growth hormone secretagogue receptor (GHSR1a) [10], is expressed and can exert endocrine/paracrine-actions at the prostate level [9]. To bind to GHSR1a, native-ghrelin must be acylated by the ghrelin-o-acyl transferase enzyme (GOAT) [11]. Intriguingly, native-ghrelin is also expressed in several cancers types including PCa [9, 12–14], suggesting a putative role in these pathologies [2, 9]. In contrast, the expression (or absence) of ghrelin receptors (GHSR1a and the truncated-variant GHSR1b) in PCa cells is still unclear [12, 13]. Despite all these data potentially linking native-ghrelin with prostate, the putative pathophysiological role of native-ghrelin in PCa has not been elucidated since some reports suggest that native-ghrelin might exert either stimulatory [14, 15] or inhibitory [16–18] effects in PCa cells proliferation/migration.

Soon after the discovery of native-ghrelin, several independent laboratories identified a number of alternative ghrelin gene-derived peptides/mRNA splice variants [2, 9, 15], which are aberrantly expressed in tumoral pathologies including PCa [9, 14, 15, 19]. Specifically, our group identified one of those variants, In1-ghrelin, which is characterized by the retention of intron-1 [20]. As a result, In1-ghrelin shares the first 13-aa with native-ghrelin, and, therefore, retains the minimum sequence required for GOAT acylation [21], whereas, the rest of its aa sequence is different, due to the retention of the intron-1. Interestingly, In1-ghrelin has been shown to be up-regulated in some endocrine-related cancers such as breast, pituitary, and neuroendocrine tumors (NETs), where it increases malignant processes such as cell proliferation/migration or hormonal secretion [20, 22, 23]. Consequently, we hypothesized that In1-ghrelin could be also present in human PCa-tissues where it could play a role in cancer-pathobiology. Hence, the central aim of this study was to investigate, for the first time, the presence, functional role and mechanisms of actions of In1-ghrelin compared with native-ghrelin in PCa by applying multiple experimental approaches including human PCa samples, preclinical mouse models, and androgen-dependent and castration-resistant PCa cell-lines.

## Methods

### Patients and samples

Fresh PCa samples (*n* = 52) obtained by core needle biopsies from patients diagnosed with palpable high-risk PCa (from which histology was confirmed by anatomo-pathologists) and normal prostate (NP) samples (*n* = 12; obtained from patients that underwent cystoprostatectomy due to bladder-cancer and confirmed as NP by the same anatomo-pathologists) were analyzed in the study (Table 1). Moreover, plasma from an additional cohort of PCa-patients with different risk levels (*n* = 30) and control-healthy patients (*n* = 20) was collected (Table 2). The study was approved by the corresponding Hospital Ethic Committee (approval number: 2461) and written informed consent was obtained from all individuals.

**Table 1:** Demographic, clinical, anatomopathological characteristics and PCR data from patients with high-risk prostate cancer (needle biopsies) and normal prostate controls (cytoprostatectomies)

Parameter	Overall	NP	PCa
Age, Median (IQR)	76 (68.2–81.7)	70 (61.5–81)	78 (69–81.7)
PSA level, ng/ml, median (IQR)			54.5 (37.2–212)
BMI, median (IQR)	27.6 (25.4–30.3)	25.8 (24.4–32.5)	27.9 (25.5–30.1)
Gleason score			
= 7	-	-	18/52 (35%)
> 7	-	-	34/52 (65%)
Extraprostatic extension	-	-	18/52 (35%)
Perineural infiltration	-	-	27/52 (52%)
% of samples where mRNA expression was detected:			
Ghrelin	56/64 (87,5%)	9/12 (83,3%)	47/52 (90%)
In1-ghrelin	54/64 (84,5%)	8/12 (66,6%)	46/52 (88,5%)
GHSR-1a	1/27 (3,7%)	0/7 (0%)	1/20 (5%)
GHSR-1b	7/28 (25,5%)	2/8 (25%)	5/20 (20%)

*NP* Normal prostate, *PCa* prostate cancer, *No* number, *EE* extraprostatic extensión, *PI* perineural infiltration

**Table 2:** Demographic and clinical characteristic of patients included in the study of plasmatic levels of In1-ghrelin and ghrelin in control (*n* = 30) and PCa patients (*n* = 20)

Parameter	Control (*n* = 20)	PCa (*n* = 30)	*p value*
Age, yr., mean (SD)	63.8 (9.9)	74.8 (8.1)	*< 0,0001*
Weight, kg, mean (SD)	82.62 (12.8)	78.7 (12.3)	0.29
BMI, mean (SD)	29.8 (4.7)	28,8 (3.6)	0.54
Total PSA, ng/ml, median (IQR)	0.85 (0.44–1.1)	25 (11.1–83.9)	*< 0,0001*
Gleason score, n (%)			
6		8/30 (26,6%)	-
7		9/30 (30%)	-
8		7/30 (23,3%)	-
9		6/30 (20%)	-
Acylated In1-ghrelin, pg/mL, median (IQR)	0 (0–0)	4.6 (0–18)	*0,03*

*PCa* prostate cancer, *Yr* year, *SD* standard desviation, *Kg* kilogram, *cm* centimeter *BMI* body mass index, *n* number, *IQR* interquartile range. *p*-values <0.05 are indicated in italics

### RNA isolation, reverse-transcription and quantitative real-time PCR (qPCR)

RNA extraction, quantification, reverse-transcription as well as the development, validation and application of qPCR to measure the expression levels of human transcripts in fresh tissue-samples and cell-lines have been previously reported [22, 24]. Briefly, expression levels (absolute mRNA copy-number/50 ng of sample) of native-ghrelin, In1-ghrelin, GHSR1a/b, Ki-67 and the housekeeping genes ACTB/GAPDH were measured using primers previously validated (primers used in the study are shown in Additional file 1: Table S1) [22, 25–27]. Expression levels in fresh PCa and NP samples were determined by qPCR and adjusted by a normalization-factor (NF) calculated from ACTB and GAPDH expression levels using GeNorm3.3-software [28]. Expression levels in prostate cell-lines were determined by qPCR and normalized according to the value of ACTB.

### Primary cultures of NP tissues

As recently reported from our group in detail [24], NP-tissue was dispersed into single-cells by enzymatic and mechanic disruption using an adapted protocol reported by Goldstein et at. [29] to obtain a cell suspension enriched in normal prostatic epithelial-cells.

### Cell-lines and reagents

Cell lines (RWPE-1, 22Rv1, LNCaP, VCaP, PC-3 and DU145) were obtained from ATCC, cultured and maintained under manufactures’ recommendations, validated by analysis of STRs (GenePrint® 10 System, Promega, Barcelona, Spain) and checked for mycoplasma contamination by PCR as previously reported [30]. Human acylated-ghrelin was commercially available (SC1357, PolyPeptide Laboratories, Limhamn, Sweden); while human acylated In1-ghrelin derived peptides (In1–19/In1–40) were synthesized in collaboration with Ipsen-Bioscience (Cambridge, MA, USA) and developed by CPC-Scientific (Chinese Peptide Company, Hangzhou, China). IGF-1 and paclitaxel peptides were used as positive or negative control in proliferation assays, respectively (Sigma-Aldrich, Madrid, Spain).

### ELISA/RIA determinations

Total-PSA (DRG-diagnostics, Marburg, Germany), acylated-ghrelin (Cat-number: EZGRA-88 K; Millipore, Madrid, Spain) and Total-Testosterone (Cat-number: ADI-900-065, Enzo-Grupo Taper, Madrid, Spain) levels were measured by ELISA following the manufactures’ instructions. For the measurement of acylated In1-ghrelin in plasma, a competitive radioimmunoassay was used (Cat-number: RK-032–42; Phoenix, Burlingame, CA, USA).

### Stable transfection of native-ghrelin and In1-ghrelin

PC-3 and VCaP cell-lines were stably transfected with pCDNA3.1 vector (Life Technologies, Madrid, Spain) containing native-ghrelin or In1-ghrelin and selected as previously reported in detail [25]. Stable transfection was confirmed by qPCR.

### Silencing of In1-ghrelin by specific siRNAs

For silencing assays, PC-3 and LNCaP cell-lines were used. Specifically, 200.000 cells were seeded in 6-well plates and grown until 70% of confluence was reached. Then, cells were individually transfected with specific siRNAs designed and previously validated against In1-ghrelin (siRNA (1) In1-ghrelin: 5′-GAGTCCTAAACAGACTGTT-3′; siRNA (2) In1-ghrelin: 5′-CACUGUUUCUGGAAGGACATT-3′) or with a scramble control (Catalog# 4390843, Invitrogen), using Lipofectamine-RNAiMAX (Invitrogen) as previously reported [22]**.** After 48 h, cells were collected for validation of the transfection (qPCR) and seeded for cell-proliferation assays and PSA-secretion.

### Functional assays: Measurements of proliferation-rate, migration and changes in free cytosolic calcium concentration ([Ca^2+^]_i_)

Cell viability/proliferation in response to native-ghrelin or In1-ghrelin overexpression and treatment was measured until 72 h by Alamar-Blue reagent (Biosource-International, Camarillo, CA, USA) or MTT-tetrazolium salt (Sigma-Aldrich, Madrid, Spain) colorimetric assays as previously reported [22, 31]. Cell-migration was evaluated by Wound-Healing assay as previously described [25]. Changes in [Ca^2+^]_i_ in response to native-ghrelin or In1-ghrelin peptides (In1–19/In1–40) in NP single cells were determined using fura-2 AM dye (50.000 cells/coverslip; Molecular-Probes, Eugene, OR) as previously reported [22].

### RT^2^ prostate-cancer PCR-Array

Total-RNA (0.75 μg) was extracted from 4 consecutive passages of mock, native-ghrelin and In1-ghrelin stably transfected PC-3-cells using Absolutely RNA RT-PCR Miniprep Kit (Agilent, La Jolla, CA, USA). Total-RNA quality was assessed using the Agilent’s 2100 Bioanalyzer (Agilent-technologies). For PCR array experiments, an RT^2^ Prostate-Cancer PCR-array was used to simultaneously examine the mRNA levels of 84 genes associated with PCa development, including five housekeeping genes (HPRT1, B2M, RPLP0, GAPDH and ACTB; used for normalization of the data) in 96-well plates, following the manufacturer’s protocol (catalog-number 330231 PAHS-135ZA, Qiagen, Limburg, Netherlands). Relative amounts were calculated by the ΔΔCt-method and further normalized to the values of their corresponding mock-samples. The resulting values were reported as fold-change. Validation of genes that showed some significant change in the array was carried out by qPCR with a different set of custom-designed primers (Additional file 1: Table S2).

### Western blotting

Two hundred fifty thousand LNCaP and PC-3 cells were cultured in 6-well plates and incubated for 24 h in complete growth-medium (supplemented with 10% FBS). Then, medium was removed and cells were starved overnight in HBSS-medium (Gibco, Madrid, Spain). Next day, medium was replaced with free FBS-growth medium and treated with native-ghrelin, In1-ghrelin (In1–19/In1–40) and IGF-1 (as positive control) peptides at 10 nM for 5, 15 and 30 min. Medium without treatment was used as vehicle-control. 500,000 PC-3-cells overexpressing In1-ghrelin, native-ghrelin and their respective control (mock) cells were cultured in 6-well plates and incubated for 24 h for the validation of the changes observed in RT2 Prostate-Cancer PCR-Array. Then, cells were washed and lysed with SDS-DTT buffer as previously reported [22, 25]. Proteins were separated by SDS-PAGE and transferred to nitrocellulose-membranes (Millipore, Madrid, Spain). Membranes were blocked with 5% non-fat dry milk in Tris-buffered saline with 0,05% Tween-20 and incubated O/N at 4C with the primary antibodies for phospho-ERK1/2 (ref: 4370), phospho-AKT (ref: 9271), anti-SFRP1 (ref: 3534), anti-APC (ref: 2504), anti-B-tubulin (ref: 2128) and anti-Total Androgen Receptor (D6F11) (ref: 5153S) from Cell Signaling (Danvers, MA, USA), anti-ZNF185 (ref: ab83100), anti-IL-6 (ref: 667) and anti-CDKN2A/p16INK4a (ref: ab81278) from Abcam (Cambridge, UK); anti-LOXL1 (ref: sc-166,632), anti-NRIP1 (ref: sc-8997), IGFBP5 (ref: sc-13,093) from Santa Cruz (CA, USA) and anti-phospho-Androgen Receptor (Ser81) (ref: 07-1375) from Millipore. Secondary anti-rabbit (ref: 7074) and anti-mouse were purchased from Cell Signaling (Danvers, MA, USA). Proteins were developed using an enhanced chemiluminescence detection-system (GE Healthcare, Little Chalfont, UK) with dyed molecular weight markers. A densitometric analysis of the bands was carried out with ImageJ software.

### Xenograft model of tumor growth

Experiments with mice were carried out following the European Regulations for Animal Care under the approval of the University/Regional’s Government Research Ethics-Committees. Ten-week-old male athymic BALB/cAnNRj-Foxn1nu mice (Janvier-Labs, Le Genest St Isle, France) were subcutaneously grafted in both flanks with 2 × 10^6^ mock, native-ghrelin or In1-ghrelin stably-transfected PC-3-cells resuspended in 100 μl of basement membrane-extract (ref:3432–005-001; Trevigen, Maryland, USA) and the tumor-growth was monitorized once per week during 3-months as previously reported [25]. Each tumor was dissected, fixed and sectioned for histopathological-examination after hematoxylin–eosin staining for the examination of necrosis, mitosis and inflammatory infiltration by three expert anatomo-pathologists. Another piece from the tumor was kept at −80 °C for RNA-extraction and quantification of expression levels (by qPCR) of genes associated with PCa-development as described above.

### Statistical analysis

Kolmogorov-Smirnov test was used to analyze the normality of the values. Parametric-data were compared by two-tailed t-test, while nonparametric-data were compared by Mann-Whitney test. Correlations were studied using Spearman-correlation test. The difference between tumor-growth in nude-mice was evaluated by two-way ANOVA. *P*-values < 0.05 were considered statistically significant. When *p*-values ranged between < 0.1 and > 0.05, a trend for significance was indicated where appropriate. All statistical analyses were performed using the GraphPad-Prism (La Jolla, CA, USA).

## Results

### In1-ghrelin, but not native-ghrelin, is overexpressed in PCa tissues and cell lines

**Fig. 1:**
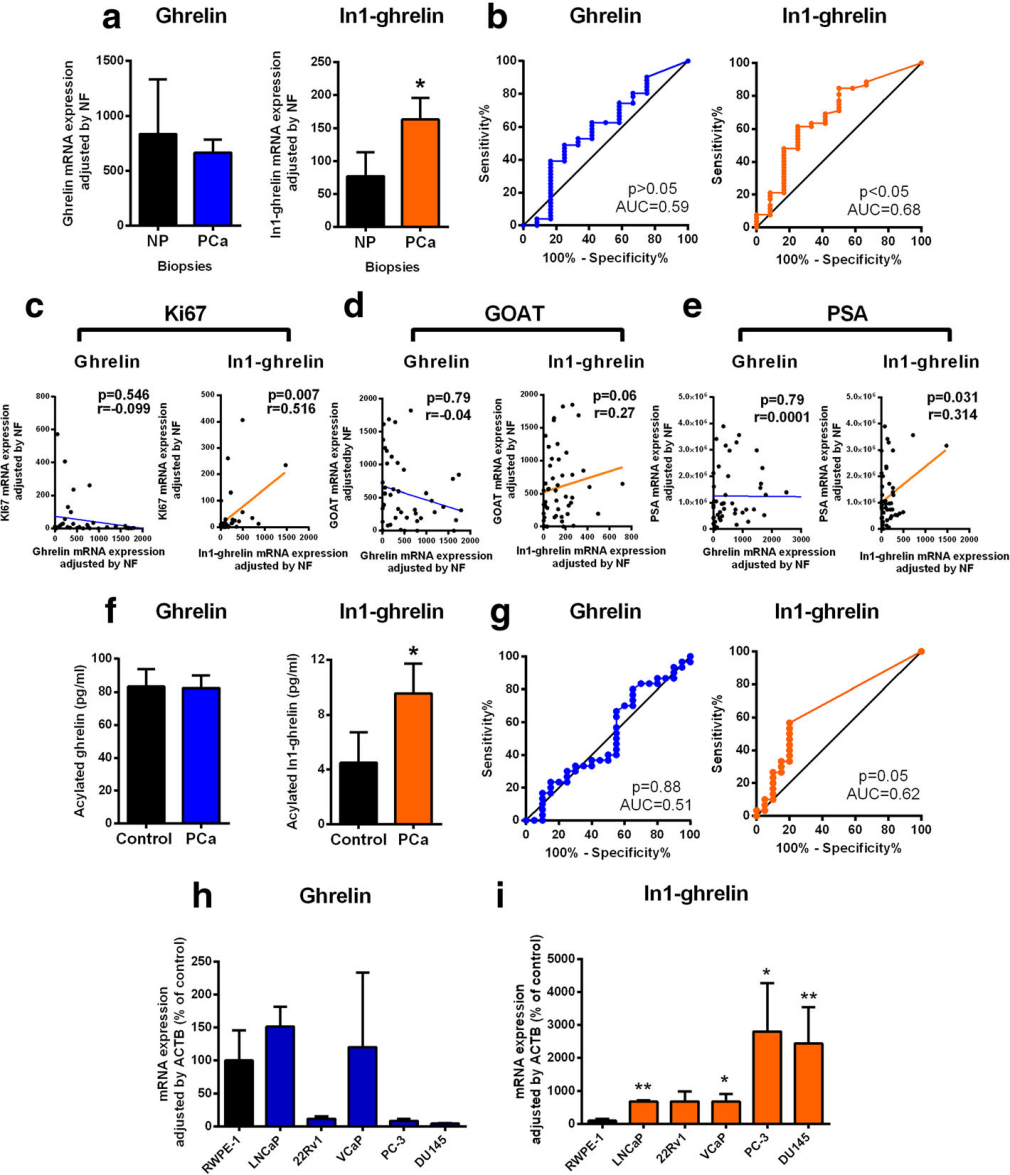
Expression of ghrelin and In1-ghrelin in prostate cancer. **a**. Ghrelin or In1-ghrelin mRNA expression in biopsies from patients with high-risk PCa (*n* = 52) and normal prostate from patients that underwent cystoprostatectomy (*n* = 12). Expression levels were determined by qPCR and adjusted by a normalization factor (NF) calculated from ACTB and GAPDH expression levels; **b**. ROC curve analysis to determine the accuracy of ghrelin or In1-ghrelin mRNA expression as diagnostic test to discriminate between high-risk PCa patients and controls using the same cohort. **c**. Correlations between ghrelin or In1-ghrelin expression with Ki-67 mRNA expression in PCa patients **d**. Correlations between ghrelin or In1-ghrelin expression with GOAT enzyme mRNA expression in PCa patients. **e**. Correlations between ghrelin or In1-ghrelin with PSA in PCa patients; **f**. Expression of acylated-ghrelin (ELISA) or acylated In1-ghrelin (RIA) in the plasma of patients with PCa (*n* = 30) and controls (*n* = 20); **g**. ROC curve analysis to determine the accuracy of acylated ghrelin or acylated In1-ghrelin plasmatic protein expression as diagnostic test to discriminate between PCa patients and controls. **h**. Ghrelin mRNA expression levels in normal-like prostate cell line (RWPE-1) or PCa cell lines; **i**. In1-ghrelin mRNA expression levels in normal-like prostate cell line (RWPE-1) or PCa cell lines. Absolute mRNA levels from different passages (*n* ≥ 3) were determined by qPCR and adjusted by ACTB. Asterisks (*p < 0.05; **p < 0.01, ***p < 0.001) indicate values that significantly differ. Data represent mean ± SEM

Analysis of the ghrelin-system in PCa biopsies from a cohort (Table 1) of high-risk patients (*n* = 52) compared to normal prostate (NP) control samples (*n* = 12), revealed that native-ghrelin and In1-ghrelin mRNA was detected in 83,3 and 66,6% of control-patients and in 90 and 88,5% of PCa samples, respectively (Table 1). However, GHSR1a expression was only detected in one tumor, while GHSR1b expression was detected in 6 controls and 10 tumor samples but its levels were negligible (Table 1; Additional file 1: Figure S1A). Remarkably, In1-ghrelin, but not native-ghrelin, mRNA was significantly overexpressed in PCa-samples (Fig. 1a). Indeed, ROC-curve analysis demonstrated that only In1-ghrelin expression could significantly discriminate between patients with or without PCa (Fig. 1b). Moreover, In1-ghrelin, but not native-ghrelin, expression was positively correlated with the expression of Ki-67 (a classic proliferation marker) (Fig. 1c), GOAT enzyme (Fig. 1d) and with PSA (Fig. 1e).

We next studied the presence and regulation of circulating levels of native-ghrelin and In1-ghrelin in an independent patient’s cohort (Table 2). Acylated In1-ghrelin was detected in plasma and its plasmatic levels, but not native-ghrelin, were significantly higher in PCa-patients (*n* = 30) compared to controls (*n* = 20) (Fig. 1f). Remarkably, ROC-curve analysis demonstrated that only acylated In1-ghrelin plasmatic levels could discriminate between patients with or without PCa (*p* = 0.05; Fig. 1g).

Evaluation of mRNA expression of ghrelin-system in androgen-dependent and castration-resistant PCa cell-lines compared with the normal-like prostate cell-line RWPE1 revealed that while native-ghrelin was expressed in normal RWPE1-cells and in LNCaP/VCaP PCa-cells (Fig. 1h), its expression was almost undetectable in the other PCa cell-lines analyzed (Fig. 1h). However, In1-ghrelin expression was commonly higher in all PCa cell-lines compared with the normal RWPE1-cells (Fig. 1i), which is consistent with the data obtained from fresh PC-samples (Fig. 1a). Moreover, In1-ghrelin expression was higher in castration-resistant cells (DU145/PC-3) compared to androgen-dependent cells (LNCaP/22Rv1/VCaP), suggesting that In1-ghrelin expression may vary through the different stages of PCa. Conversely, GHSR1a/b were barely expressed in these PCa cell-lines (Additional file 1: Figure S1B), which is in contrast with previous studies showing the presence of GHSR1a in some of these PCa cell-lines [12]. Therefore, we used different primers sets [including a primer set used in the previously mentioned work [12]], but again we did not find any evidence of detectable GHSR1a expression in PCa cell-lines (Additional file 1: Figure S1C).

### In1-ghrelin and native-ghrelin effects on NP cell-cultures

**Fig. 2:**
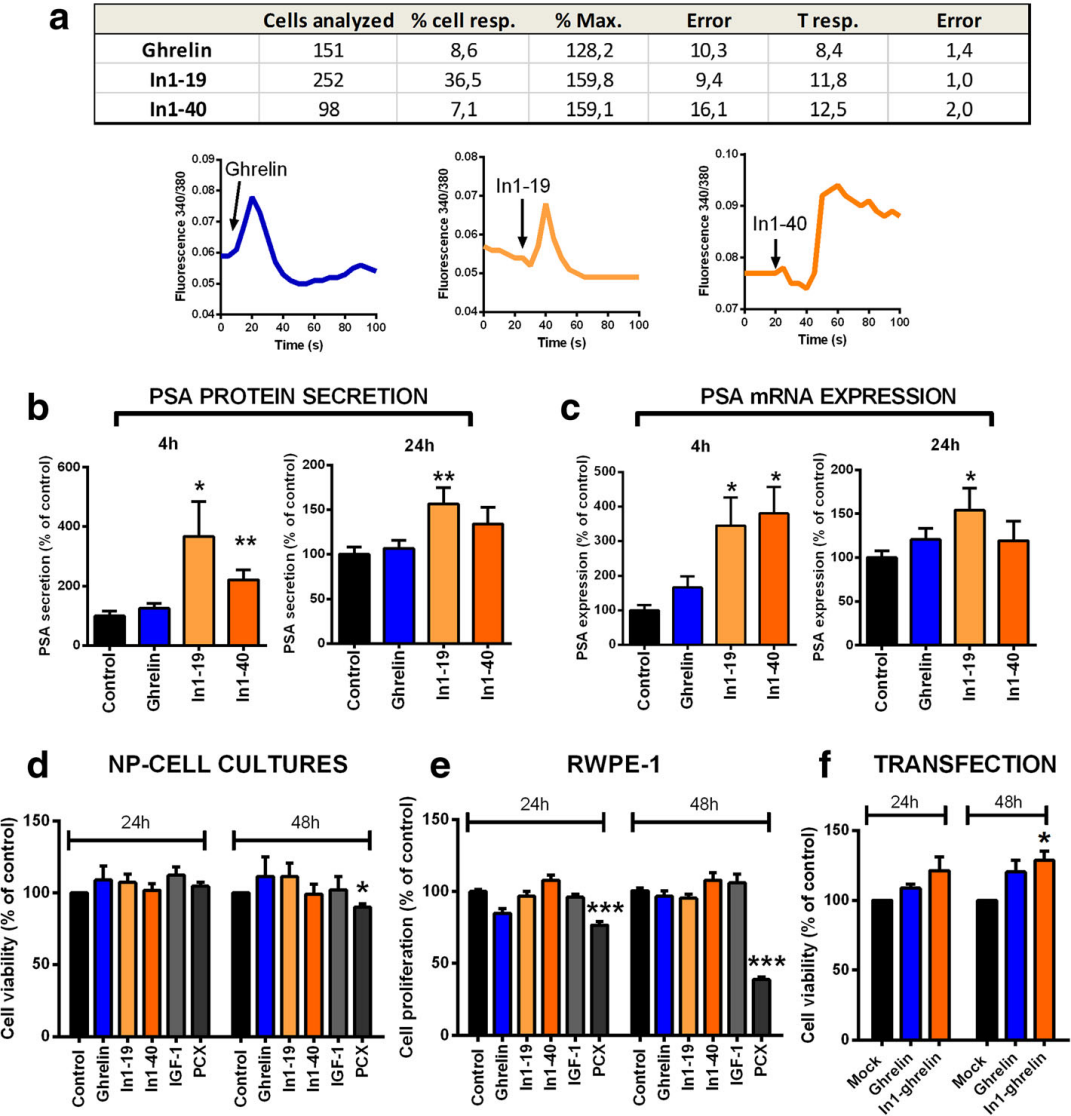
Effects of ghrelin and In1-ghrelin on normal prostate cell functions. **a**. Ghrelin and In1-ghrelin derived peptides (In1–19 and In1–40) actions on free cytosolic calcium levels ([Ca2+]i) in normal prostate single-cell from primary cultures (10 nM; *n* ≥ 3). Total number of cells measured, percentage of responsive cells (% of cells resp.), percentage of maximum response (±error) and time of maximal response (±error) are indicated. Representative profiles of changes in [Ca^2+^]i in response to ghrelin, In1–19 and In1–40 are also depicted; **b**. PSA protein secretion after 4 or 24 h of treatment with ghrelin or In1-ghrelin derived peptides (10 nM) in primary normal prostate cell cultures; **c**. PSA mRNA expression after 4 or 24 h of treatment with ghrelin or In1-ghrelin derived peptides (10 nM) in primary normal prostate cell cultures; **d**. Cell viability of normal prostate cell cultures after treatment with vehicle-control, ghrelin or In1-ghrelin derived peptides for 4-24 h (*n* ≥ 3); **e**. Cell proliferation of normal prostate RWPE-1 cell line after treatment with vehicle-control, ghrelin or In1-ghrelin derived peptides for 4-24 h (*n* ≥ 3). **f**. Cell viability of normal prostate cell cultures transfected with empty (mock), ghrelin or In1-ghrelin vectors and determined after 24-48 h (*n* ≥ 3). *Asterisk* represent significant differences (*p < 0.05; ***p < 0.001) between control and ghrelin or In1-ghrelin effects (treatment or transfection). Data represent mean ± SEM

Calcium signaling is one of the intracellular mechanisms involved on prostate cell physiology, where it triggers hormone secretion and has thus been used to evaluate prostate cell function [32, 33]. Therefore, changes in free-cytosolic calcium concentration ([Ca^2+^]_i_) in single cells derived from primary NP cell-cultures were analyzed in response to In1-ghrelin derived peptides (In1–19 and In1–40) and native-ghrelin (Fig. 2a). We found that native-ghrelin and In1-ghrelin could induce a stimulatory response in [Ca^2+^]_i_ in NP-cells however, the percentage of responsive cells was different upon treatment (i.e. whereas 36,5% of NP-cells responded to In1–19 treatment, only 8,6% and 7,1% responded to native-ghrelin or to In1–40, respectively). Moreover, In1-ghrelin peptides evoked a higher stimulatory response compared with native-ghrelin (i.e. an increase of 58% and 59% in response to In1–19 and In1–40 vs. an increase of 28% with native-ghrelin).

Remarkably, In1-ghrelin treatment, but not native-ghrelin, increased PSA (a prostate-specific marker associated to PCa progression) protein secretion (Fig. 2b) and mRNA expression (Fig. 2c) in NP-cell cultures at 4 h and 24 h, being In1–19 the most effective peptide (Fig. 2b, c). Remarkably, these effects are clearly higher at 4 h (with a 3–4-fold increase in PSA secretion/expression in comparison with the control), than at 24 h (with a 1.5-fold increase in PSA secretion/expression), which may suggest a predominantly acute, short-term effect of In1-ghrelin peptides on PSA synthesis/secretion in NP cells. Moreover, native-ghrelin or In1-ghrelin derived peptides treatment did not affect cell-viability in primary NP-cell cultures (Fig. 2d) or in normal RWPE1-cells (Fig. 2e). However, In1-ghrelin, but not native-ghrelin, overexpression increased the cell-viability of primary NP-cell cultures compared to mock (control)-transfected cell cultures (Fig. 2f). In this sense, it should be noted that this discrepancy between overexpression and treatment experiments in NP cells could be related to the different dynamics of the experimental approaches, wherein In1-ghrelin treatment represents an acute, short-term effect that decline over time, while In1-ghrelin overexpression plausibly maintains peptide levels over time, and therefore, it likely provides a better model to observe an effect on cell viability in response to In1-ghrelin overexposure.

### In1-ghrelin peptides treatment increased malignant features of PCa cells

**Fig. 3:**
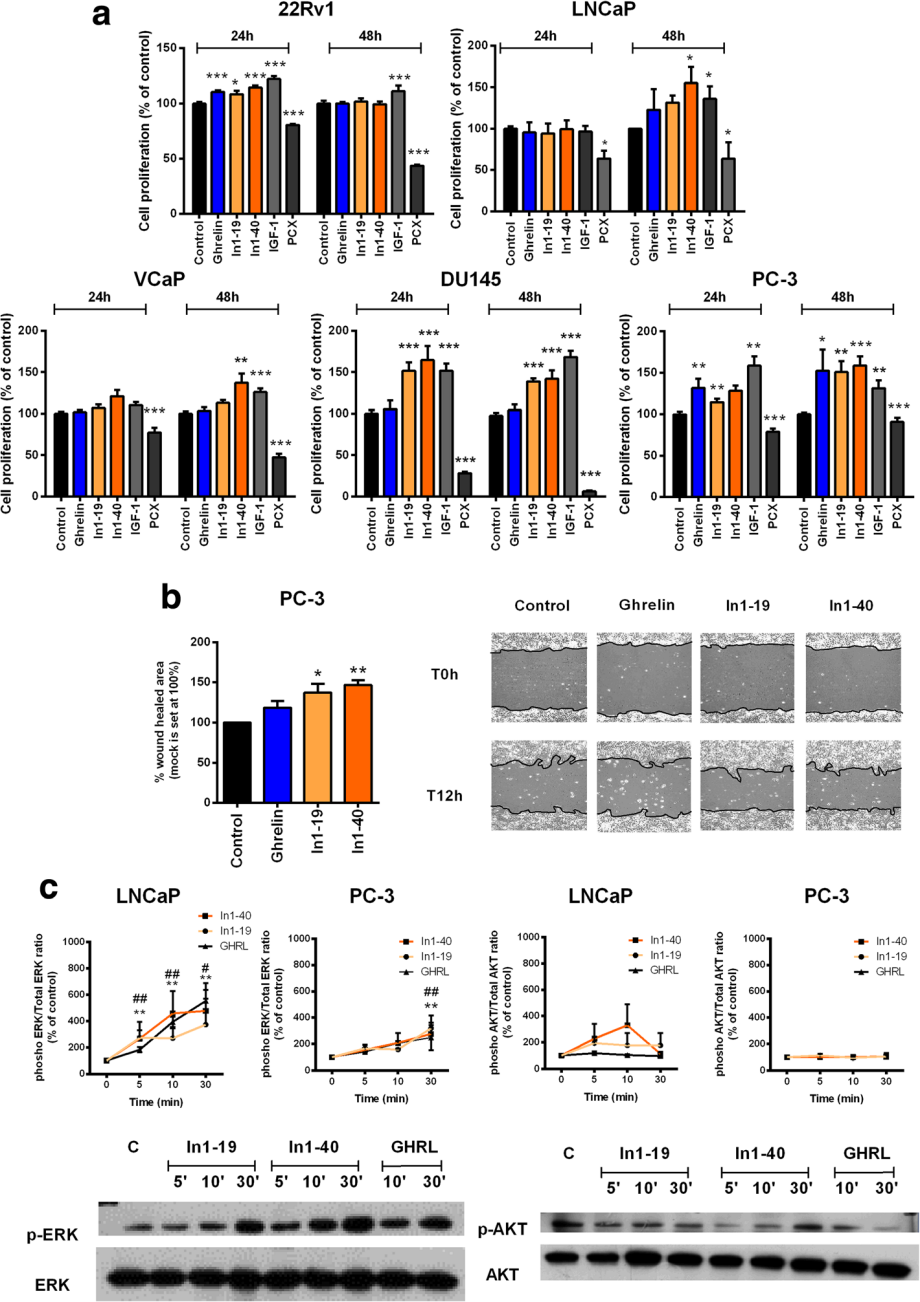
Effects of ghrelin and In1-ghrelin on PCa pathophysiological processes. **a**. Cell proliferation of PCa cell lines after treatment with ghrelin or In1-ghrelin derived peptides for 4-24 h (10 nM; *n* ≥ 3). Treatment with IGF-1 and PCX were used as positive and negative controls, respectively. **b**. Effect of ghrelin and In1-ghrelin peptides treatment on the migration PC-3 cell line was determined by wound healing assay (12 h; *n* ≥ 3). Representative images showing the higher migration capacity of PC-3 cells after treatment with In1-ghrelin peptides are depicted. **c**. phospho-ERK and phospho-AKT time-course activation after treatment with ghrelin or In1-ghrelin peptides (5–30 min) in LNCaP and PC-3 cell lines. Protein levels of phospho-ERK and phospho-AKT were adjusted by total ERK and AKT, respectively. Data represent mean ± SEM. *Asterisks* (***p < 0.001, **p < 0.01, *p < 0.05) indicate differences between In1–19 and vehicle-treated controls, and dashes between In1–40 and vehicle control treatment (# < 0.05; ##*p* < 0.01). Representative blots in LNCaP cell line are showed

Administration of native-ghrelin only increased cell proliferation of 22Rv1 (at 24 h) and PC-3 (24-48 h) cell lines, whereas it did not exert any significant effect in the rest of PCa cell lines studied (i.e. LNCaP, VCaP and DU145; Fig. 3a). However, In1-ghrelin peptides were able to increase cell proliferation in several PCa cell lines, both androgen-dependent (22Rv1 VCaP and LNCaP) and castration- resistant cells (PC-3/DU145) (Fig. 3a). Indeed, the effect of In1-ghrelin peptides treatment was more consistent and pronounced in castration-resistant PCa cell lines (PC-3 and DU145), especially at 24 h in DU145 and at 48 h in PC-3 (Fig. 3a). However, in androgen-dependent PCa cell lines, both peptides increased proliferation at 24 h in 22Rv1 cells, while only In1–40 increased proliferation at 48 h in LNCaP and VCaP cells (Fig. 3a). In1-ghrelin peptides, but not native-ghrelin, were able to increase migration of PC-3 cells (Fig. 3b). Finally, In1-ghrelin peptides, and ghrelin peptide, induced the phosphorylation on ERK1/2 in PC-3 and LNCaP cell lines (Fig. 3c), with almost no detectable effect on AKT-phosphorylation (Fig. 3c). Remarkably, In1-ghrelin or native-ghrelin peptides were not able to alter basal testosterone secretion from LNCaP cells (Additional file 1: Figure S2A). Similarly, treatment with In1-ghrelin and native-ghrelin peptides did not alter AR expression or phosphorylation (Additional file 1: Figure S2B, C), as well as PSA expression and/or secretion on LNCaP cells (Additional file 1: Figure S2D), suggesting that the effects attributed to In1-ghrelin peptides may not be mediated by the modulation of androgen/AR system.

### In1-ghrelin overexpression increased proliferation/migration of PCa cell lines

**Fig. 4:**
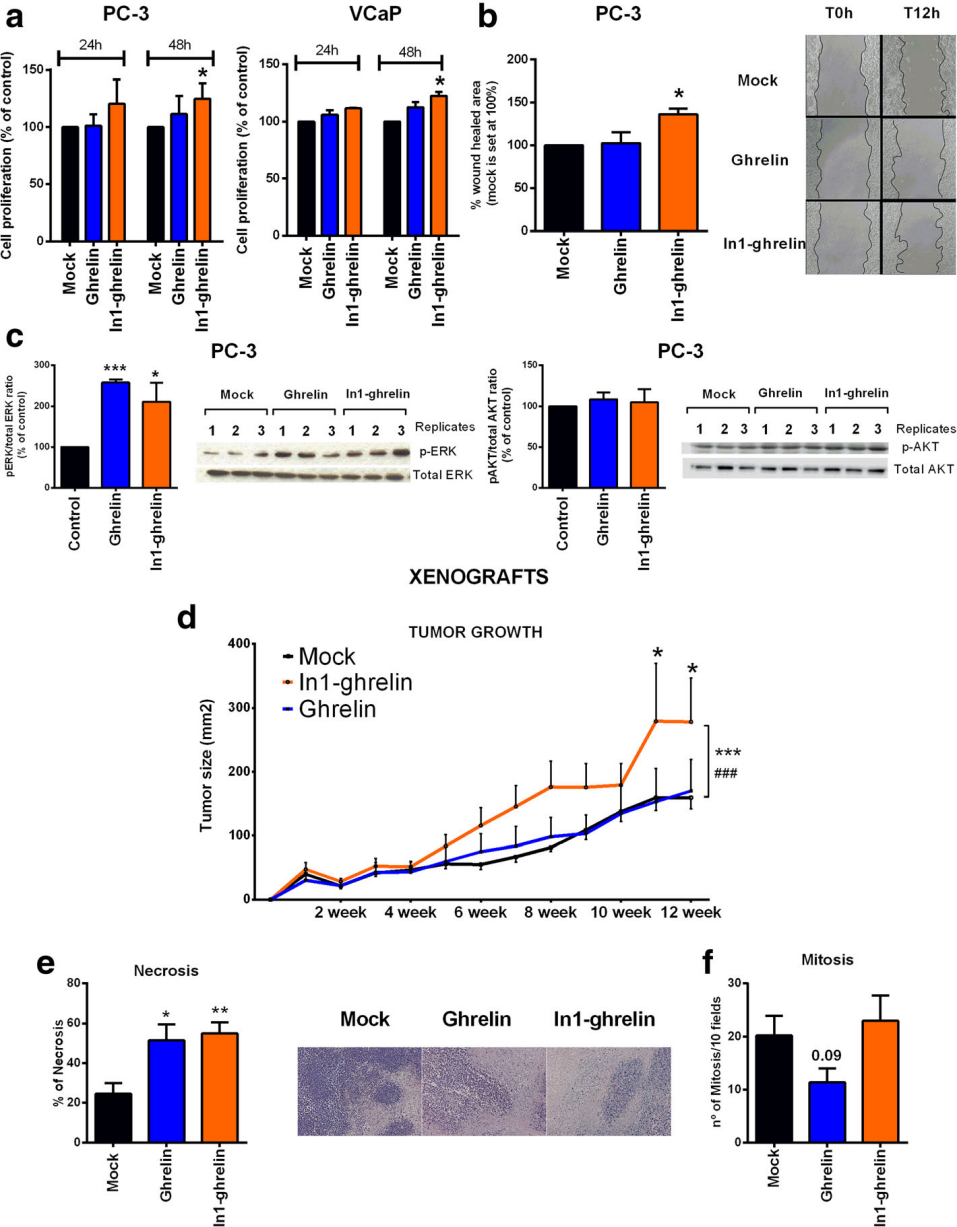
Effect of ghrelin and In1-ghrelin overexpression on PCa pathophysiological processes. **a**. Cell proliferation of empty (mock), ghrelin and In1-ghrelin vectors stably transfected PC-3 and VCaP cell lines for 24-48 h (*n* ≥ 3); **b**. Cell migration of mock, ghrelin and In1-ghrelin stably transfected PC-3 cell line after 12 h by wound-healing assay (*n* ≥ 3). Representative images showing the migration capacity of PC-3 cells transfected with mock, ghrelin and In1-ghelin are also indicated; **c**. phospho-ERK and phospho-AKT basal activation in PC-3 stably transfected cells (*n* ≥ 3). Blots are representative of one cell passage with three technical replicates; **d**. Growth rate of subcutaneously inoculated mock, ghrelin and In1-ghrelin-transfected PC-3-derived tumors in nude mice (*n* = 5) followed up to 12 weeks after inoculation. Statistical significance was evaluated by two-way ANOVA (***p < 0.001 indicate differences between In1-ghrelin and mock, while #*p* < 0.001 indicates differences between In1-ghrelin and ghrelin stably transfected cells); **e**. % of necrosis in xenografted PC-3-derived tumors. Representative images of hematoxylin–eosin (H/E) staining are depicted. **f**. N° of mitosis/10 fields in xenografted PC-3-derived tumors. Data represent mean ± SEM (*n* ≥ 3). *Asterisks* (***p < 0.001; **p < 0.01; *p < 0.05) indicate values that significantly differ from the mock control

In1-ghrelin, but not native-ghrelin, overexpression increased cell proliferation in androgen-dependent (VCaP) and castration-resistant (PC-3) cell lines at 48 h (Fig. 4a). Efficiency of stable transfection of In1-ghrelin or ghrelin was confirmed by qPCR (Additional file 1: Figure S3). Moreover, In1-ghrelin, but not native-ghrelin overexpression increased the migration of PC-3 cells (Fig. 4b). Strikingly, overexpression of native-ghrelin and In1-ghrelin increased the basal-phosphorylation of ERK, with no changes in Akt-phosphorylation (Fig. 4c).

### In1-ghrelin overexpression enhanced the growth rate of PC-3 induced xenografted tumors

Consistent with in vitro data, subcutaneous tumors induced by In1-ghrelin overexpression in a preclinical model (engrafted stably-transfected PC-3 cells in the flanks of immunodeficient mice) were larger than those induced with mock or native-ghrelin-transfected cells (*p* = 0.0001 in both cases; Fig. 4d). Moreover, histological analysis revealed that tumors with stably-transfected native-ghrelin and In1-ghrelin cells showed a higher necrosis grade (Fig. 4e), without changes in percentage of mitotic cells (Fig. 4f), compared to mock-induced tumors.

### In1-ghrelin overexpression evoked a profound dysregulation of key genes involved in PCa development/progression

**Fig. 5:**
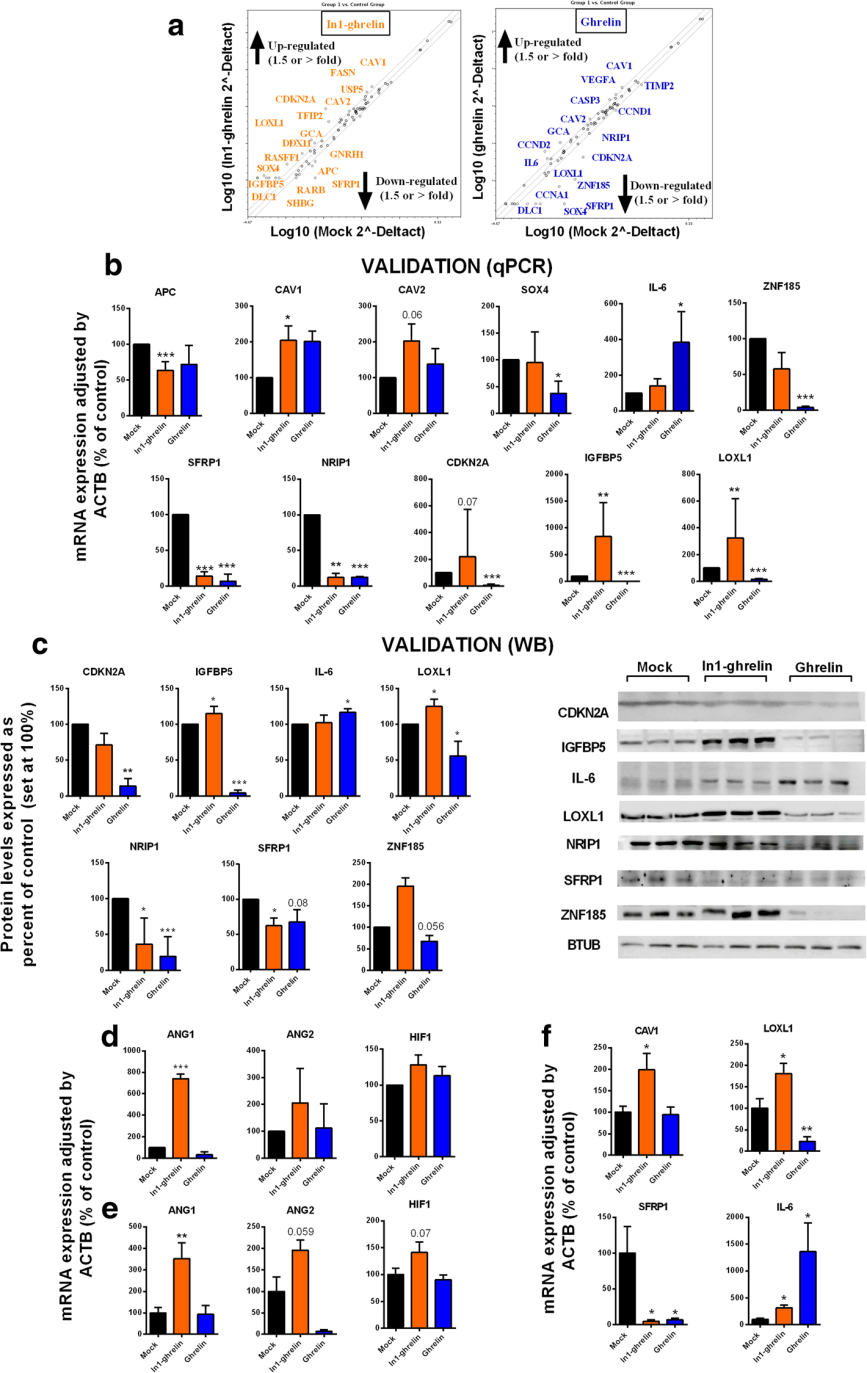
Gene expression effects of ghrelin and In1-ghrelin overexpression in PC-3 and derived xenografted tumors. **a**. Results from the RT2 Prostate-Cancer PCR Array which evaluates the expression of 84 genes involved in prostate cancer development and progression performed in ghrelin and In1-ghrelin-stably transfected PC-3-cells compared with control-mock PC-3-cells. The graphs indicate those genes which expression change ≥ 1.5-fold; **b**. Validation by qPCR of genes dysregulated in the RT2 Prostate-Cancer PCR Array using different cell preparations (*n* ≥ 3) and new sets of primers; **c**. Validation by Western blot of the changes observed in the previous analysis; **d**. Expression of angiogenic factors in In1-ghrelin-stably transfected PC-3-cells and native ghrelin-stably transfected PC-3 cells compared with control-mock PC-3-cells.; **e**. Expression of angiogenic factors in xenografted tumors of stably transfected-PC-3 cells. **f**. CAV1, LOXL1, SFRP1 and IL-6 mRNA expression levels in mock, ghrelin and In1-ghrelin transfected PC-3-derived xenografted tumors. Results were normalized with ACTB. All preparations were repeated at least three times (*n* ≥ 3). (*p < 0.05; **p < 0.01; ***p < 0.001). Values represent mean (±SEM) or median (IQR). *Asterisks* (***p < 0.001; **p < 0.01; *p < 0.05) indicate values that significantly differ from the mock control

In order to uncover the molecular changes induced by In1-ghrelin overexpression in PCa, we performed a qPCR array comprising 84 key genes involved in PCa development/progression (Fig. 5a and, Additional file 1: Table S2). We found 18 genes whose expression was altered more than 1.5-fold in In1-ghrelin stably-transfected PC-3-cells (13 up-regulated: CAV1, CAV2, CDKN2A, DDX11, DLC1, FASN, GCA, IGFBP5, LOXL1, RASSF, SOX4, TFPI2 and USP5; and, 5 down-regulated: APC, GNRH1, RARB, SFRP1 and SHBG; Fig. 5a), as well as 17 genes whose expression was altered in native-ghrelin stably-transfected PC-3-cells (7 up-regulated: CASP3, CAV1, CAV2, CCND2, GCA, IL-6 and VEGFA; and, 10 down-regulated: CCNA1, CCND1, CDKN2A, DLC1, LOXL1, NRIP1, SFRP1, SOX4, TIMP2 and ZNF185; Fig. 5a). Next, we used qPCR analysis using cDNA from different passages of native-ghrelin and In1-ghrelin stably-transfected PC-3-cells and different sets of primers (Additional file 1: Table S2) to validate the results of the array. Thus, we observed that CAV1, CAV2, CDKN2A, IGFBP5 and LOXL1 were upregulated while APC, NRIP1 and SFRP1 were downregulated in In1-ghrelin stably-transfected PC-3-cells (Fig. 5b). Similarly, we confirmed that IL-6 was up-regulated while CDKN2A, IGFBP5, LOXL1, NRIP1, SFRP1, SOX4 and ZNF185 were downregulated in native-ghrelin stably-transfected PC-3 (Fig. 5b). Additionally, we confirmed some of these changes at the protein level [i.e. up-regulation of IGFBP5 and LOXL1 and downregulation of NRIP1 and SFRP1 in In1-ghrelin, and upregulation of IL-6 and downregulation of CDKN2A, IGFBP5, LOXL1, NRIP1, SFRP1 and ZNF185 in native-ghrelin stably-transfected PC-3 cells (Fig. 5c)].

Interestingly, some of these changes in mRNA and protein expression (Fig. 5b, c) were similar in the In1-ghrelin and native-ghrelin stably-transfected PC-3-cells (e.g. SFRP1/NRIP1 downregulation); but, most noteworthy, that some of these changes were regulated oppositely in both PCa cell-models (i.e. downregulation in native-ghrelin and up-regulation in In1-ghrelin stably-transfected PC-3-cells of LOXL1/IGFBP5; Fig. 5b, c). Altogether, these findings are reminiscent of the similar vs. disparate effects observed previously with native-ghrelin and In1-ghrelin in PCa-cells, respectively (Figs. 3 and 4). Remarkably, In1-ghrelin stably-transfected PC-3-cells showed an overall increase in the expression of proangiogenic-factors (i.e. ANG1, ANG2 and HIF1) compared to mock- and native-ghrelin stably-transfected PC-3 cells (Fig. 5d; being these differences only statistically significant for ANG1). Similar results were observed on the In1-ghrelin stably-transfected PC-3 derived xenografted-tumors (Fig. 5e). Some of the changes observed in the qPCR-array, real-time qPCR, and/or western-blot analyses, such as those observed for CAV1, LOXL1, IL-6 and SFRP1 were also further validated in the xenografted-tumors (Fig. 5f). Interestingly, we found a higher inflammatory cell-infiltration in the native-ghrelin, but not In1-ghrelin, stably-transfected PC-3-tumors (Additional file 1: Figure S4) which, together with the increase in IL-6 expression, suggest a role of native-ghrelin in tumor inflammation.

### In1-ghrelin silencing decreased cell proliferation and PSA secretion

**Fig. 6:**
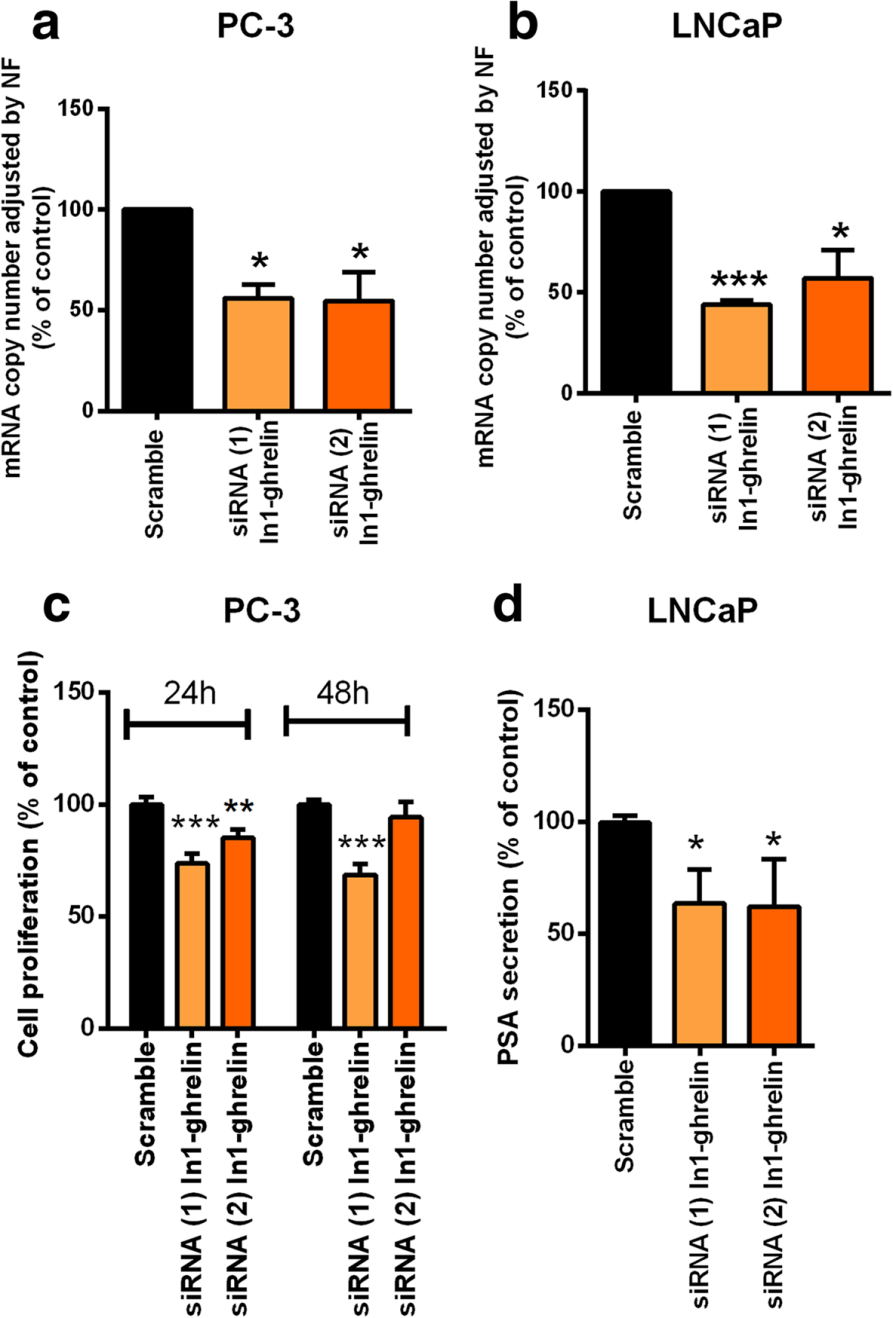
Effects of In1-ghrelin silencing on PCa cell proliferation and PSA secretion. **a**. Validation by qPCR of In1-ghrelin silencing in PC-3; **b**. Validation by qPCR of In1-ghrelin silencing in LNCaP cells. In both cases, expression levels were adjusted by a normalization factor (NF) calculated from ACTB and GAPDH expression levels; **c**. Proliferation rates of In1-ghrelin-silenced PC-3 and LNCaP cells compared with control scramble-transfected cells; **d**. PSA secretion of In1-ghrelin-silenced LNCaP cells compared with control scramble-transfected cells. All experiments were repeated at least three times (*n* ≥ 3). Data were evaluated by two-tailed t-test to analyze significant differences (*p < 0.05; **p < 0.01, ***p < 0.001) and represent mean ± SEM and are expressed as percentage vs control (scramble-treated cells), which was set at 100%

Downregulation of In1-ghrelin expression using two specific In1-ghrelin siRNAs, which was validated by qPCR (Fig. 6a, b) and did not implied compensatory changes in native ghrelin expression (Additional file 1: Figure S5), decreased cell proliferation in PC-3 and LNCaP cell-lines at 24 h and/or 48 h [Fig. 6c; siRNA-2 tended to decrease cell-proliferation at 48 h in LNCaP-cells (*p* = 0.06) but this difference did not reach statistical significance]. Moreover, In1-ghrelin silencing significantly decreased PSA secretion in LNCaP cell line using both siRNAs (Fig. 6d).

## Discussion

Previous studies have shown that native-ghrelin is expressed in both NP and PCa tissues/cell-lines with an increased staining of ghrelin-peptide in malignant prostate epithelium compared with normal glandular-tissue [14]. Interestingly, additional reports have shown that other ghrelin-gene derived splicing variants are also present in PCa where they could be involved in PCa malignancy [2, 14, 15]. Herein, we have expanded those results by demonstrating that In1-ghrelin mRNA levels are overexpressed in a battery of PCa biopsies from patients diagnosed with high-risk PCa, compared to NP samples, which is consistent with previous results indicating that In1-ghrelin overexpression is a common hallmark shared by other endocrine-related tumors such as breast-cancer, pituitary-tumors and NETs [20, 22, 23]. However, although the expression of native-ghrelin was higher than that of In1-ghrelin in NPs, in our study cohort, native-ghrelin mRNA levels were not significantly elevated in PCa-samples. Indeed, In1-ghrelin, but not ghrelin levels, levels were directly correlated with those of Ki-67 (a classical proliferation marker previously found to be correlated with In1-ghrelin expression in breast cancer samples [20]), and ROC-curve analysis revealed that only In1-ghrelin expression (but not native-ghrelin) could discriminate between patients with or without PCa, suggesting that In1-ghrelin merits further study as a potential novel biomarker in PCa. Interestingly, In1-ghrelin, but not native-ghrelin, levels positively correlated with GOAT-expression in PCa, an association that has also been previously found in other endocrine-related tumors [20, 22, 23], and suggests that In1-ghrelin may be the main ghrelin-gene variant functionally linked to GOAT in those tumors, which also reinforces the idea that an autocrine/paracrine-circuit involving these two components of the ghrelin-system may operate in PCa. Indeed, this association might be particularly relevant in PCa pathology because we have recently demonstrated that GOAT-enzyme is overexpressed in PCa patients and its levels exhibit high specificity/sensitivity to predict PCa presence compared with other PCa biomarkers [24].

Remarkably, analysis carried out in an additional PCa patient’s cohort and controls demonstrated that acylated In1-ghrelin peptide could be detected in plasma and that these levels, but not those of native-ghrelin, are significantly higher in PCa patients than in healthy-controls, which again would suggest the possible utility of In1-ghrelin levels as a novel biomarker for PCa patients by using non-invasive (liquid) biopsies. Moreover, consistent with the results found in PCa biopsies, In1-ghrelin mRNA expression was higher in PCa cell lines compared with the NP cell-line. Interestingly, we observed that In1-ghrelin, but not native-ghrelin, expression seemed to be higher in castration-resistant than in those androgen-dependent cell lines, which would suggest that In1-ghrelin might play a role in the late PCa stages and/or in the progression of the disease.

The fact that In1-ghrelin, but also native-ghrelin, were expressed at substantial levels in NP- and PCa-cells suggested that they could be exerting a functional role in the normal and pathological physiology of the prostate. Therefore, NP cell-cultures were used to test whether In1-ghrelin peptides and native-ghrelin can modulate signaling and functional-parameters (such as PSA-production and cell-viability) in these cells. Indeed, In1-ghrelin peptides and native-ghrelin treatment evoked a signaling response in terms of changes in the [Ca^2+^]_i_, a key second messenger that has been previously linked to ghrelin-system signaling [22, 34] and is functionally associated to PCa-pathophysiology. Specifically, it has been shown that ion-channel remodeling alters the nature of PCa-cells Ca^2+^ influx, switching from an apoptotic to a pro-proliferative stage [35]. Nevertheless, it should be noted that the proportion of responsive cells and the magnitude of the stimulatory increase were consistently higher after In1-ghrelin than after native-ghrelin treatment in all experiments performed on primary NP-cells. This observation suggests that In1-ghrelin variant could be playing a more pronounced function than native-ghrelin in prostate cells. Nonetheless, our results demonstrate a direct action of ghrelin-system splicing variants on NP-cells, which would imply the existence of receptors for In1-ghrelin, but also for native-ghrelin, in these cells. However, such putative receptors, likely, ought to be different from the classical GHSR1a, as we found that GHSR1a expression is virtually absent in the human prostate samples and PCa cell lines analyzed herein. The existence of uncharacterized receptor(s) that mediate some of the biological effects of native-ghrelin, and likely of In1-ghrelin, has also been previously postulated in human prostate neoplasms and related cell-lines [13]. Interestingly, In1-ghrelin, but not native-ghrelin overexpression clearly increased cell-viability, and In1-ghrelin treatment also increased PSA-secretion/expression in NP cell-cultures, which is consistent with data previously reported indicating that In1-ghrelin treatment enhances hormone-secretion in other cell-types [i.e. serotonin in NETs cells [23], GH in somatotropinoma cells and, ACTH in corticotropinoma cells [22]] and may suggest a relevant role of In1-ghrelin in the malignization of NP-cells.

Remarkably, ghrelin-gene derived variants, especially the In1-ghrelin variant, exerted a relevant effect on the pathophysiology of PCa cells. Indeed, In1-ghrelin treatment evoked an increase in the proliferation on most of the PCa cell lines tested, being its effect particularly marked in castration-resistant cell lines. Similarly, In1-ghrelin peptides significantly enhanced the migration capacity of PC-3 cells. The ability of In1-ghrelin peptides to increase cell proliferation has been previously reported in human pituitary tumors (and in mouse AtT-20 cell-line) [22]. However, native-ghrelin administration only increased cell proliferation in LNCaP and PC-3 cell lines, which is in agreement with previous studies [14]. These actions of In1-ghrelin peptides, and also ghrelin peptide as previously published [14], may be mediated, at least in part, through the activation of ERK signaling pathways, as previously shown in other tumoral pathologies [14, 22] and do not seem to be mediated by the modulation of the androgen/AR system. Consistent with these data, stable overexpression of In1-ghrelin, but not native-ghrelin enhanced cell proliferation and migration capacity of PCa cells (PC-3 and VCaP cells). In line with this, In1-ghrelin overexpression has been shown to increase the cell-proliferation of pituitary-tumors [22], MDA-MB-231 breast-cancer cell-line [20] and BON-1 pancreatic cell-line [23], and the migration capacity of NETs cell lines [23]. Moreover, to further explore this notion, we generated, for the first time, nude mice injected with In1-ghrelin or with native-ghrelin stably-transfected PC-3 cells and found that In1-ghrelin, but not native-ghrelin, overexpression enhanced tumors-growth in this in vivo preclinical-model (i.e. larger tumors), wherein it increased tumor-necrosis, likely due to higher tumor volume supporting the idea that In1-ghrelin would increase the malignant features of PCa cells.

To gain further insight on the mechanisms underlying the actions of the ghrelin-variants on PCa cells, we explored changes in the expression of a set of selected genes related to PCa. Specifically, In1-ghrelin increased malignant-features of PCa cells by altering the expression of key-oncogenes, tumor-suppressor genes and genes associated to PCa pathophysiology such as APC, CAV1, SFRP1, NRIP1, CDKN2A, IGFBP5 and LOXL1 [36–41], which could help to explain the functional changes triggered by In1-ghrelin over-exposition (treatment and/or overexpression). Indeed, some changes were further confirmed at the protein-level (i.e. SFRP1, NRIP1, IGFBP5 and LOXL1) and in the In1-ghrelin stably-transfected PC-3 tumors, which showed an increase in CAV1 and LOXL1, and a decreased in SFRP1, expression compared to mock cell-induced tumors. Intriguingly, native-ghrelin treatment and/or overexpression elicited the phosphorylation of ERK-pathway in PCa cells and induced significant changes in the expression of certain oncogenes, tumor-suppressor genes and in genes associated to PCa pathophysiology. Some of these changes were different (i.E. *il*-6, SOX4 and ZNF185), common (i.e. decreased in SFRP1 and NRIP1) or opposite (i.e. LOXL1 and IGFBP5) to those observed by In1-ghrelin overexpression. Our study demonstrates that native-ghrelin can also display significant effects on the functional endpoints measured (in vitro proliferation and/or migration and in vivo proliferation and inflammation), although they are more reduced compared to In1-ghrelin effects, suggesting that native-ghrelin could be playing a role on other endpoints not measured herein, and that In1-ghrelin spliced variant has a more relevant role than native-ghrelin in the aggressiveness of PCa cells. In line with this, the increase in LOXL1 and IGFBP5 expression observed in PCa cells overexpressing In1-ghrelin could be pathophysiological relevant and could be associated to the unique capacity of In1-ghrelin to enhance the malignancy-features in PCa cells, since both factors have been shown to increase the aggressiveness of PCa-cells [40–43]. Specifically, LOXL1 is able to enhance tumorigenesis and metastasis through active remodeling of tumor-microenvironment [40, 42]. Moreover, IGFBP5 has been shown to play an important role in the castration-phase of the disease, since upregulation in its expression accelerates progression to androgen-independence in PCa models [43], and enhances proliferation of PCa cells [41]. Accordingly, it is tempting to speculate that the upregulation of LOXL1 and IGFBP5 observed in PCa-cells overexpressing In1-ghrelin might be associated to the increased aggressiveness-features observed in PCa cells (i.e. in vitro and/or in vivo cell proliferation, migration, tumor growth and PSA secretion).

Finally, we further explored the utility of In1-ghrelin as a putative target to reduce PCa progression by analyzing the effect of In1-ghrelin silencing on PCa cell lines functional parameters. Remarkably, In1-ghrelin silencing decreased cell-proliferation of PC-3 and LNCaP cells, and PSA secretion from LNCaP cells, which, overall suggest that In1-ghrelin could be considered as a novel target for the development of new and more specific therapies in PCa.

## Conclusions

Thus, when viewed together, our results indicate that In1-ghrelin splicing variant is overexpressed in PCa, where it can regulate cell proliferation, migration, tumor growth and PSA secretion, through the modulation of the activation of certain signaling-pathways (ERK phosphorylation) and the expression of several oncogenes and tumor-suppressor genes, thereby suggesting a possible pathophysiological role for this splice-variant in human PCa. The fact that the ghrelin-system, particularly its In1-ghrelin variant, was strongly altered in PCa supports the idea that this system could contribute to PCa tumorigenesis, and may provide novel tools to explore diagnostic/therapeutic-targets in this pathology.

## Additional file

Additional file 1:
**Figure S1.** Ghrelin receptor expression in PCa. **A**. GHSR1a or GHSR1b mRNA expression in biopsies from patients with high-risk PCa (*n* = 20) and normal prostate samples from patients that underwent cystoprostatectomies (*n* = 7). Expression levels were determined by qPCR and adjusted by a normalization factor (NF) calculated from ACTB and GAPDH expression levels. Data were evaluated by Mann-Whitney test and represent median (IQR); **B**. GHSR1a or GHSR1b mRNA expression in PCa cell lines (androgen-dependent: VCaP, 22Rv1 and castration resistant: PC-3, DU145). Absolute mRNA levels from different passages (*n* ≥ 3) were determined by qPCR and adjusted by ACTB [Data are expressed as percent of DU145 (GHSR1a) and VCaP (GHSR1b) set at 100%, since they are the cell lines with lesser expression of those receptors, and, in order to ease the comparison between the cell lines); **C**. Further analysis of GHSR1a expression on different PCa cell lines (22Rv1, DU145, LNCaP, PC-3), normal-like prostate cell line (EPN) and PCa samples using several pairs of primers. As positive control, cDNA from a pituitary tumor with GHSR1a expression was used. **Figure S2.** Secretion of testosterone and modulation of androgen receptor (AR) system after treatment with ghrelin gene-derived peptides in LNCaP cells. **A**. Testosterone levels in the culture medium after 24 h of treatment with native-ghrelin or In1-ghrelin peptides (10 nM); **B**. AR mRNA expression after 24 h of treatment with ghrelin or In1-ghrelin derived peptides (10 nM); **C**. phospho-AR (Ser81) time-course activation after treatment with native-ghrelin or In1-ghrelin peptides (10–30 min). Protein levels of phospho-AR (Ser81) were adjusted by total AR. Representative blots in LNCaP cell line are showed; **D**. PSA secretion and mRNA expression after 24 h of treatment with native-ghrelin or In1-ghrelin peptides (10 nM). Absolute mRNA levels were determined by qPCR and adjusted by ACTB. Values represent mean ± SEM of *n* > 3 experiments. **Figure S3.** Representative figures showing the validation of In1-ghrelin and ghrelin overexpression in PC3 cells by qPCR. Absolute mRNA levels were determined by qPCR and adjusted by ACTB. Values represent mean ± SEM of *n* > 3 experiments. Asterisk indicate significant difference (****p* < 0.001). **Figure S4.** Grade of inflammation in xenografted PC3-derived tumors. Representative images of hematoxylin–eosin (H/E) staining are depicted. Tumors derived from native ghrelin stably-transfected PC-3 cells presents more inflammation (observed as darker foci in the pictures) compared to In1-ghrelin or mock derived tumors**.** Asterisk indicate significant difference (**p* < 0.05). **Figure S5.** Expression of native-ghrelin in LNCaP and PC-3 cells in response to In1-ghrelin silencing by specific siRNA. Absolute mRNA levels were determined by qPCR and adjusted by ACTB. Values represent mean ± SEM of *n* > 3 experiments. **Table S1.** List of primers used in the studies. **Table S2.** Prostate cancer finder RT^2^ Profiler PCR array data. (ZIP 2150 kb)
