# Supplementary material for: The oncogenic role of the In1-ghrelin splicing variant in prostate cancer aggressiveness
Source: Mol Cancer. 2017 Aug 29;16:146. doi: 10.1186/s12943-017-0713-9 (PMC5576296; doi:10.1186/s12943-017-0713-9)
Supplement: Additional file 1: — Figure S1. Ghrelin receptor expression in PCa. A. GHSR1a or GHSR1b mRNA expression in biopsies from patients with high-risk PCa (n = 20) and normal prostate samples from patients that underwent cystoprostatectomies (n = 7). Expression levels were determined by qPCR and adjusted by a normalization factor (NF) calculated from ACTB and GAPDH expression levels. Data were evaluated by Mann-Whitney test and represent median (IQR); B. GHSR1a or GHSR1b mRNA expression in PCa cell lines (androgen-dependent: VCaP, 22Rv1 and castration resistant: PC-3, DU145). Absolute mRNA levels from different passages (n ≥ 3) were determined by qPCR and adjusted by ACTB [Data are expressed as percent of DU145 (GHSR1a) and VCaP (GHSR1b) set at 100%, since they are the cell lines with lesser expression of those receptors, and, in order to ease the comparison between the cell lines); C. Further analysis of GHSR1a expression on different PCa cell lines (22Rv1, DU145, LNCaP, PC-3), normal-like prostate cell line (EPN) and PCa samples using several pairs of primers. As positive control, cDNA from a pituitary tumor with GHSR1a expression was used. Figure S2. Secretion of testosterone and modulation of androgen receptor (AR) system after treatment with ghrelin gene-derived peptides in LNCaP cells. A. Testosterone levels in the culture medium after 24 h of treatment with native-ghrelin or In1-ghrelin peptides (10 nM); B. AR mRNA expression after 24 h of treatment with ghrelin or In1-ghrelin derived peptides (10 nM); C. phospho-AR (Ser81) time-course activation after treatment with native-ghrelin or In1-ghrelin peptides (10–30 min). Protein levels of phospho-AR (Ser81) were adjusted by total AR. Representative blots in LNCaP cell line are showed; D. PSA secretion and mRNA expression after 24 h of treatment with native-ghrelin or In1-ghrelin peptides (10 nM). Absolute mRNA levels were determined by qPCR and adjusted by ACTB. Values represent mean ± SEM of n > 3 experiments. Figure S3. Representative f [file 12943_2017_713_MOESM1_ESM.zip › 12943_2017_713_MOESM1_ESM.docx]

**Additional file: Figure legends**

**Figure S1**. **Ghrelin receptor expression in PCa. A**. GHSR1a or GHSR1b mRNA expression in biopsies from patients with high-risk PCa (n=20) and normal prostate samples from patients that underwent cystoprostatectomies (n=7). Expression levels were determined by qPCR and adjusted by a normalization factor (NF) calculated from ACTB and GAPDH expression levels. Data were evaluated by Mann-Whitney test and represent median (IQR); **B.** GHSR1a or GHSR1b mRNA expression in PCa cell lines (androgen-dependent: VCaP, 22Rv1 and castration resistant: PC-3, DU145). Absolute mRNA levels from different passages (n≥3) were determined by qPCR and adjusted by ACTB [Data are expressed as percent of DU145 (GHSR1a) and VCaP (GHSR1b) set at 100%, since they are the cell lines with lesser expression of those receptors, and, in order to ease the comparison between the cell lines); **C.** Further analysis of GHSR1a expression on different PCa cell lines (22Rv1, DU145, LNCaP, PC-3), normal-like prostate cell line (EPN) and PCa samples using several pairs of primers. As positive control, cDNA from a pituitary tumor with GHSR1a expression was used.

**Figure S2**: **Secretion of testosterone and modulation of androgen receptor (AR) system after treatment with ghrelin gene-derived peptides in LNCaP cells**. **A**. Testosterone levels in the culture medium after 24h of treatment with native-ghrelin or In1-ghrelin peptides (10nM); **B.** AR mRNA expression after 24h of treatment with ghrelin or In1-ghrelin derived peptides (10nM); **C**. phospho-AR (Ser81) time-course activation after treatment with native-ghrelin or In1-ghrelin peptides (10-30 min). Protein levels of phospho-AR (Ser81) were adjusted by total AR. Representative blots in LNCaP cell line are showed; **D**. PSA secretion and mRNA expression after 24h of treatment with native-ghrelin or In1-ghrelin peptides (10nM). Absolute mRNA levels were determined by qPCR and adjusted by ACTB. Values represent mean ± SEM of n>3 experiments.

**Figure S3**: **Representative figures showing the validation of In1-ghrelin and ghrelin overexpression in PC3 cells by qPCR**. Absolute mRNA levels were determined by qPCR and adjusted by ACTB. Values represent mean ± SEM of n>3 experiments. Asterisk indicate significant difference (***p<0.001).

**Figure S4. Grade of inflammation in xenografted PC3-derived tumors.** Representative images of hematoxylin–eosin (H/E) staining are depicted. Tumors derived from native ghrelin stably-transfected PC-3 cells presents more inflammation (observed as darker foci in the pictures) compared to In1-ghrelin or mock derived tumors**.** Asterisk indicate significant difference (*p<0.05).

**Figure S5. Expression of native-ghrelin in LNCaP and PC-3 cells in response to In1-ghrelin silencing by specific siRNA.** Absolute mRNA levels were determined by qPCR and adjusted by ACTB. Values represent mean ± SEM of n>3 experiments**.**

**Table S1. Prostate cancer finder RT^2^ Profiler PCR array data**

|  |  |  | **Fold change** | |
| --- | --- | --- | --- | --- |
| **Gene symbol** | **Official full name** | **Refseq** | **In1-ghrelin** | **Ghrelin** |
| **ACACA** | **Acetyl-CoA carboxylase alpha** | **NM_198834** | **-1,23** | **-1,13** |
| **AKT1** | **V-akt murine thymoma viral oncogene homolog 1** | **NM_005163** | **1,24** | **-1,04** |
| **APC** | **Adenomatous polyposis coli** | **NM_000038** | **-2,02** | **-1,22** |
| **AR** | **Androgen receptor** | **NM_000044** | **NE** | **NE** |
| **ARNTL** | **Aryl hydrocarbon receptor nuclear translocator-like** | **NM_001178** | **-1,45** | **1,34** |
| **BCL2** | **B-cell CLL/lymphoma 2** | **NM_000633** | **1,13** | **-1** |
| **CAMKK1** | **Calcium/calmodulin-dependent protein kinase kinase 1, alpha** | **NM_032294** | **-1,48** | **-1,17** |
| **CAMSAP1** | **Calmodulin regulated spectrin-associated protein 1** | **NM_015447** | **-1,03** | **-1,09** |
| **CASP3** | **Caspase 3, apoptosis-related cysteine peptidase** | **NM_004346** | **-1,08** | **1,67** |
| **CAV1** | **Caveolin 1, caveolae protein, 22kDa** | **NM_001753** | **1,83** | **1,66** |
| **CAV2** | **Caveolin 2** | **NM_001233** | **1,7** | **1,65** |
| **CCNA1** | **Cyclin A1** | **NM_003914** | **1,72** | **-2,77** |
| **CCND1** | **Cyclin D1** | **NM_053056** | **-1,08** | **-1,66** |
| **CCND2** | **Cyclin D2** | **NM_001759** | **-1,33** | **3,25** |
| **CDH1** | **Cadherin 1, type 1, E-cadherin (epithelial)** | **NM_004360** | **NE** | **NE** |
| **CDKN2A** | **Cyclin-dependent kinase inhibitor 2A (melanoma, p16, inhibits CDK4)** | **NM_000077** | **7,75** | **-10,2** |
| **CLN3** | **Ceroid-lipofuscinosis, neuronal 3** | **NM_000086** | **1,07** | **-1,13** |
| **CREB1** | **CAMP responsive element binding protein 1** | **NM_004379** | **1,32** | **1,39** |
| **DAXX** | **Death-domain associated protein** | **NM_001350** | **1,42** | **1,13** |
| **DDX11** | **DEAD/H (Asp-Glu-Ala-Asp/His) box polypeptide 11** | **NM_004399** | **1,76** | **1,12** |
| **DKK3** | **Dickkopf homolog 3 (Xenopus laevis)** | **NM_015881** | **NE** | **NE** |
| **DLC1** | **Deleted in liver cancer 1** | **NM_006094** | **3,17** | **-1,51** |
| **ECT2** | **Epithelial cell transforming sequence 2 oncogene** | **NM_018098** | **1,5** | **1,28** |
| **EDNRB** | **Endothelin receptor type B** | **NM_000115** | **NE** | **NE** |
| **EGFR** | **Epidermal growth factor receptor** | **NM_005228** | **-1,19** | **1,01** |
| **EGR3** | **Early growth response 3** | **NM_004430** | **-1,05** | **1,27** |
| **ERG** | **V-ets erythroblastosis virus E26 oncogene homolog (avian)** | **NM_182918** | **NE** | **NE** |
| **ETV1** | **Ets variant 1** | **NM_004956** | **-1,41** | **-1,27** |
| **FASN** | **Fatty acid synthase** | **NM_004104** | **1,81** | **-1,28** |
| **FOXO1** | **Forkhead box O1** | **NM_002015** | **-1,5** | **-1,48** |
| **GCA** | **Grancalcin, EF-hand calcium binding protein** | **NM_012198** | **2,65** | **2,28** |
| **GNRH1** | **Gonadotropin-releasing hormone 1 (luteinizing-releasing hormone)** | **NM_000825** | **-1,85** | **1,04** |
| **GPX3** | **Glutathione peroxidase 3 (plasma)** | **NM_002084** | **NE** | **NE** |
| **GSTP1** | **Glutathione S-transferase pi 1** | **NM_000852** | **-1,03** | **1,08** |
| **HAL** | **Histidine ammonia-lyase** | **NM_002108** | **NE** | **NE** |
| **HMGCR** | **3-hydroxy-3-methylglutaryl-CoA reductase** | **NM_000859** | **-1,11** | **-1,34** |
| **IGF1** | **Insulin-like growth factor 1 (somatomedin C)** | **NM_000618** | **NE** | **NE** |
| **IGFBP5** | **Insulin-like growth factor binding protein 5** | **NM_000599** | **5,01** | **-1,1** |
| **IL6** | **Interleukin 6 (interferon, beta 2)** | **NM_000600** | **-1,19** | **1,84** |
| **KLHL13** | **Kelch-like 13 (Drosophila)** | **NM_033495** | **NE** | **NE** |
| **KLK3** | **Kallikrein-related peptidase 3** | **NM_001648** | **NE** | **NE** |
| **LGALS4** | **Lectin, galactoside-binding, soluble, 4** | **NM_006149** | **NE** | **NE** |
| **LOXL1** | **Lysyl oxidase-like 1** | **NM_005576** | **7,28** | **-5,9** |
| **MAPK1** | **Mitogen-activated protein kinase 1** | **NM_002745** | **1,01** | **-1** |
| **MAX** | **MYC associated factor X** | **NM_002382** | **-1** | **-1,07** |
| **MGMT** | **O-6-methylguanine-DNA methyltransferase** | **NM_002412** | **1,1** | **1,01** |
| **MKI67** | **Antigen identified by monoclonal antibody Ki-67** | **NM_002417** | **1,02** | **-1,13** |
| **MSX1** | **Msh homeobox 1** | **NM_002448** | **1,31** | **1,4** |
| **MTO1** | **Mitochondrial translation optimization 1 homolog (S. cerevisiae)** | **NM_012123** | **-1,1** | **1,27** |
| **NDRG3** | **NDRG family member 3** | **NM_022477** | **-1,12** | **-1,25** |
| **NFKB1** | **Nuclear factor of kappa light polypeptide gene enhancer in B-cells 1** | **NM_003998** | **-1,08** | **1,01** |
| **NKX3-1** | **NK3 homeobox 1** | **NM_006167** | **-1,05** | **1,29** |
| **NRIP1** | **Nuclear receptor interacting protein 1** | **NM_003489** | **-1,26** | **-4,26** |
| **PDLIM4** | **PDZ and LIM domain 4** | **NM_003687** | **1,5** | **1,18** |
| **PDPK1** | **3-phosphoinositide dependent protein kinase-1** | **NM_002613** | **1,04** | **1,04** |
| **PES1** | **Pescadillo homolog 1, containing BRCT domain (zebrafish)** | **NM_014303** | **1,14** | **1,01** |
| **PPP2R1B** | **Protein phosphatase 2, regulatory subunit A, beta** | **NM_002716** | **1,05** | **-1,01** |
| **PRKAB1** | **Protein kinase, AMP-activated, beta 1 non-catalytic subunit** | **NM_006253** | **-1,07** | **1,01** |
| **PTEN** | **Phosphatase and tensin homolog** | **NM_000314** | **NE** | **NE** |
| **PTGS1** | **Prostaglandin-endoperoxide synthase 1 (prostaglandin G/H synthase and cyclooxygenase)** | **NM_000962** | **NE** | **NE** |
| **PTGS2** | **Prostaglandin-endoperoxide synthase 2 (prostaglandin G/H synthase and cyclooxygenase)** | **NM_000963** | **NE** | **NE** |
| **RARB** | **Retinoic acid receptor, beta** | **NM_000965** | **-1,6** | **-1,35** |
| **RASSF1** | **Ras association (RalGDS/AF-6) domain family member 1** | **NM_007182** | **1,98** | **1,43** |
| **RBM39** | **RNA binding motif protein 39** | **NM_004902** | **-1,23** | **-1,06** |
| **SCAF11** | **SR-related CTD-associated factor 11** | **NM_004719** | **-1,24** | **-1,23** |
| **sep-07** | **Septin 7** | **NM_001788** | **-1,22** | **-1,14** |
| **SFRP1** | **Secreted frizzled-related protein 1** | **NM_003012** | **-5,16** | **-24,42** |
| **SHBG** | **Sex hormone-binding globulin** | **NM_001040** | **-1,64** | **-1,18** |
| **SLC5A8** | **Solute carrier family 5 (iodide transporter), member 8** | **NM_145913** | **NE** | **NE** |
| **SOCS3** | **Suppressor of cytokine signaling 3** | **NM_003955** | **-1,11** | **1,08** |
| **SOX4** | **SRY (sex determining region Y)-box 4** | **NM_003107** | **2,5** | **-4,47** |
| **SREBF1** | **Sterol regulatory element binding transcription factor 1** | **NM_004176** | **1,43** | **-1,41** |
| **STK11** | **Serine/threonine kinase 11** | **NM_000455** | **-1,4** | **1,2** |
| **SUPT7L** | **Suppressor of Ty 7 (S. cerevisiae)-like** | **NM_014860** | **-1,33** | **1** |
| **TFPI2** | **Tissue factor pathway inhibitor 2** | **NM_006528** | **2,88** | **-1,39** |
| **TGFB1I1** | **Transforming growth factor beta 1 induced transcript 1** | **NM_015927** | **-1,05** | **1** |
| **TIMP2** | **TIMP metallopeptidase inhibitor 2** | **NM_003255** | **-1,33** | **-1,58** |
| **TIMP3** | **TIMP metallopeptidase inhibitor 3** | **NM_000362** | **NE** | **NE** |
| **TMPRSS2** | **Transmembrane protease, serine 2** | **NM_005656** | **NE** | **NE** |
| **TNFRSF10D** | **Tumor necrosis factor receptor superfamily, member 10d, decoy with truncated death domain** | **NM_003840** | **-1,12** | **-1,06** |
| **TP53** | **Tumor protein p53** | **NM_000546** | **-1** | **1,12** |
| **USP5** | **Ubiquitin specific peptidase 5 (isopeptidase T)** | **NM_003481** | **1,61** | **1,01** |
| **VEGFA** | **Vascular endothelial growth factor A** | **NM_003376** | **-1,05** | **1,6** |
| **ZNF185** | **Zinc finger protein 185 (LIM domain)** | **NM_007150** | **1,41** | **-6,23** |

| **Template** | **GenBank Accession** | **Sense** | **Antisense** | **Product length (bp)** |
| --- | --- | --- | --- | --- |
| **ACTB** | NM_001101 | ACTCTTCCAGCCTTCCTTCCT | CAGTGATCTCCTTCTGCATCCT | 176 |
| **ANG1** | NM_001146.3 | GACAGATGTTGAGACCCAGGTA | TCTCTAGCTTGTAGGTGGATAATGAA | 89 |
| **ANG2** | NM_001147.2 | GGATGGAGACAACGACAAATG | GGACCACATGCATCAAACC | 78 |
| **APC** | NM_000038 | GAACCAAGGTGGAAATGGTG | AAAGCTGGATGAGGAGAGGAA | 153 |
| **CAV1** | NM_001753 | CGACCCTAAACACCTCAACG | CAGCAAGCGGTAAAACCAGT | 148 |
| **CAV2** | NM_001233 | ACGACTCCTACAGCCACCAC | CAGCTTGAGATGCGAGTTGA | 107 |
| **CDKN2A** | NM_000077 | ACCAGAGGCAGTAACCATGC | ACCTTCGGTGACTGATGATCTAA | 121 |
| **GAPDH** | NM_002046.5 | AATCCCATCACCATCTTCCA | AATCCCATCACCATCTTCCA | 122 |
| **GHRL** | NM_016362.3 | CACCAGAGAGTCCAGCAGAGA | CCGGACTTCCAGTTCATC | 215 |
| **HIF1A** | NM_001530.3 | TTAGATTTTGGCAGCAACGAC | GGGTGAGGGGAGCATTACA | 87 |
| **IGFBP5** | NM_000599 | TGTGACCGCAAAGGATTCTAC | AAAGTCCCCGTCAACGTACTC | 129 |
| **IN1-GHRL** | GU942497.1 | TCTGGGCTTCAGTCTTCTCC | GCTTGGCTGGTGGCTTCTT | 132 |
| **IL6** | NM_000600 | GGCAGAAAACAACCTGAACCT | CTCAAACTCCAAAAGACCAGTGA | 115 |
| **KI67** | NM_002417 | GACATCCGTATCCAGCTTCCT | GCCGTACAGGCTCATCAATAAC | 139 |
| **KLK3** | NM_001030047 | GTGCTTGTGGCCTCTCGT | CAGCAAGATCACGCTTTTGT | 108 |
| **LOXL1** | NM_005576 | CATTACCACAGCATGGACGA | GCCCTGGGTATGAGAGGTG | 159 |
| **MBOAT4** | NM_001100916.1 | TTGCTCTTTTTCCCTGCTCTC | ACTGCCACGTTTAGGCATTCT | 161 |
| **NRIP1** | NM_003489 | TCACAGGTCACAGCCAAAGA | GGGCGAGAAGCATTATTTCC | 110 |
| **SFRP1** | NM_003012 | CATGCAGTTCTTCGGCTTCT | GTTGTCACAGGGAGGACACAC | 145 |
| **SOX4** | NM_003107 | TGGCTGACTACCCCGACTAC | ACCGACCTTGTCTCCCTTCT | 119 |
| **VEGFA** | NM_001171623.1 | TTAAACGAACGTACTTGCAGATG | GAGAGATCTGGTTCCCGAAA | 93 |
| **ZNF185** | NM_007150 | CTGGCTACAAGATGACCACTGA | CCTCTGACCTCCGTTTCTGTT | 144 |

**Table S2. List of primers used in the studies**
